# Supplementary material for: Forming a three-dimensional porous organic network via solid-state explosion of organic single crystals
Source: Nat Commun. 2017 Nov 17;8:1599. doi: 10.1038/s41467-017-01568-3 (PMC5693943; doi:10.1038/s41467-017-01568-3)
Supplement: Supplementary file 1 — Supplementary Information [file 41467_2017_1568_MOESM1_ESM.pdf]

Supplementary Figures

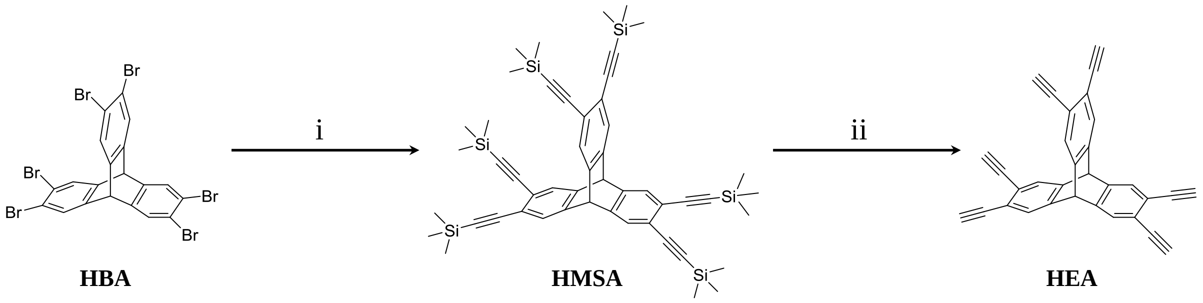

Supplementary Figure 1 | Synthesis of HEA from HBA. Reaction conditions. (i) Trimethylsilylacetylene, CuI, $PdCl_2(PPh_3)_2$, PPh_3, i-Pr_2NH; (ii) NaOH/MeOH, CH_2Cl_2.

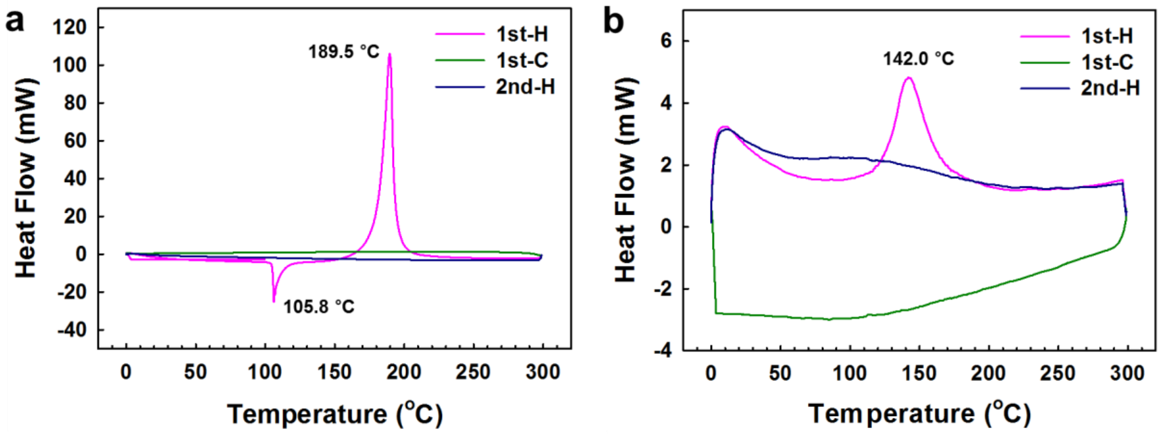

Supplementary Figure 2 | DSC thermograms obtained with a ramping rate of 10 °C min^{-1} under nitrogen atmosphere. a, Triethynylbenzene crystals, showing a typical endothermic melting peak at 105.8 °C followed by an exothermic reaction peak at 189.5 °C; **b,** Ground HEA crystals, showing only an exothermic reaction peak at 142.0 °C without showing a melting peak.

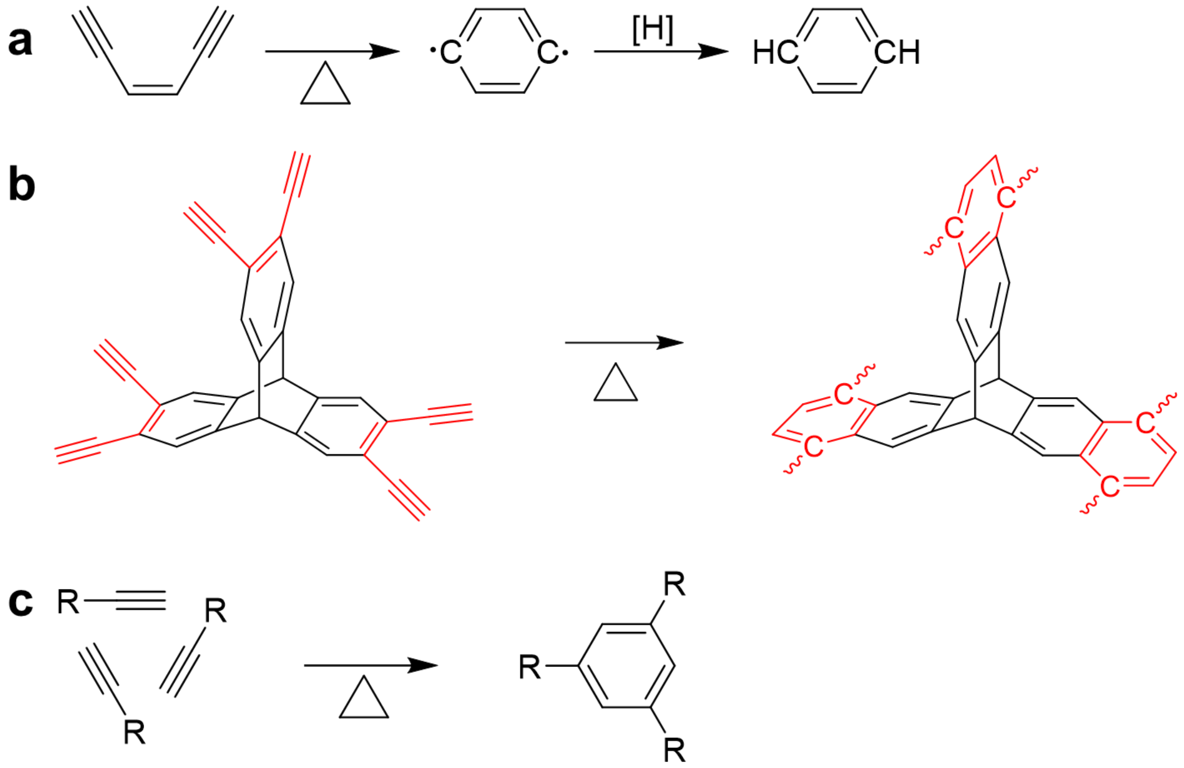

Supplementary Figure 3 | Reaction mechanism of ethynyl groups and corresponding products. a, The Bergman reaction from the simplest enediyne molecule; **b,** the Bergman reaction of HEA; **c,** the formation of one phenyl ring after cycloaromatization of three ethynyl groups.

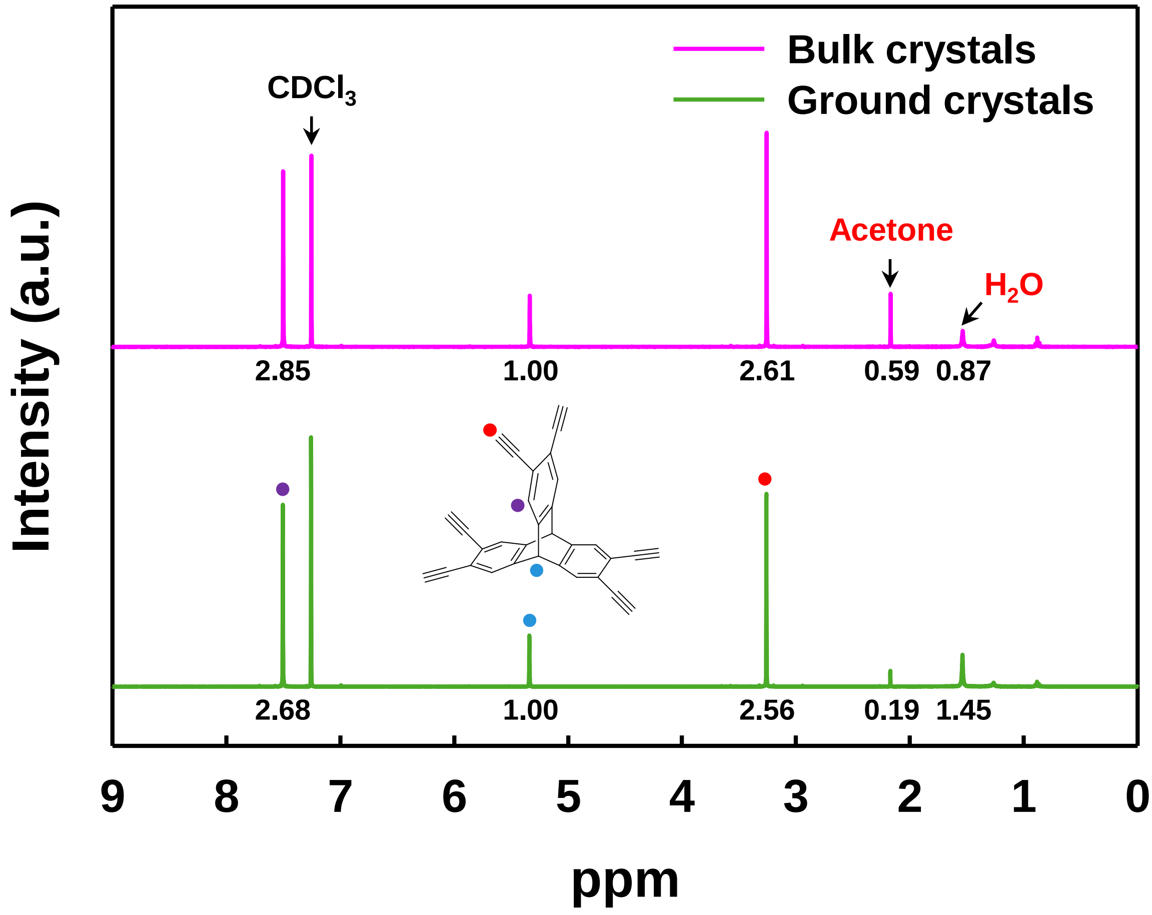

Supplementary Figure 4 | ^{1}H-NMR spectra of bulk and ground HEA crystals. After grinding, the amount of acetone decreased, while the quantity of water increased due to large surface area for more moisture uptake in air: (top) 400 MHz ^{1}H NMR of bulk crystals ($CDCl_3$, δ = ppm): 1.538 (0.87, H_2O), 2.171 (0.59, CH_3-C(=O)-CH_3), 3.260 (2.61,≡C-*H*), 5.341 (1.00, C*H*), 7.508 (2.85, Ar-*H*), (bottom) 400 MHz ^{1}H NMR of ground crystals ($CDCl_3$, δ = ppm): 1.537 (1.45, H_2O), 2.171 (0.19, CH_3-C(=O)-CH_3), 3.259 (2.56,≡C-*H*), 5.342 (1.00, C*H*), 7.508 (2.68, Ar-*H*).

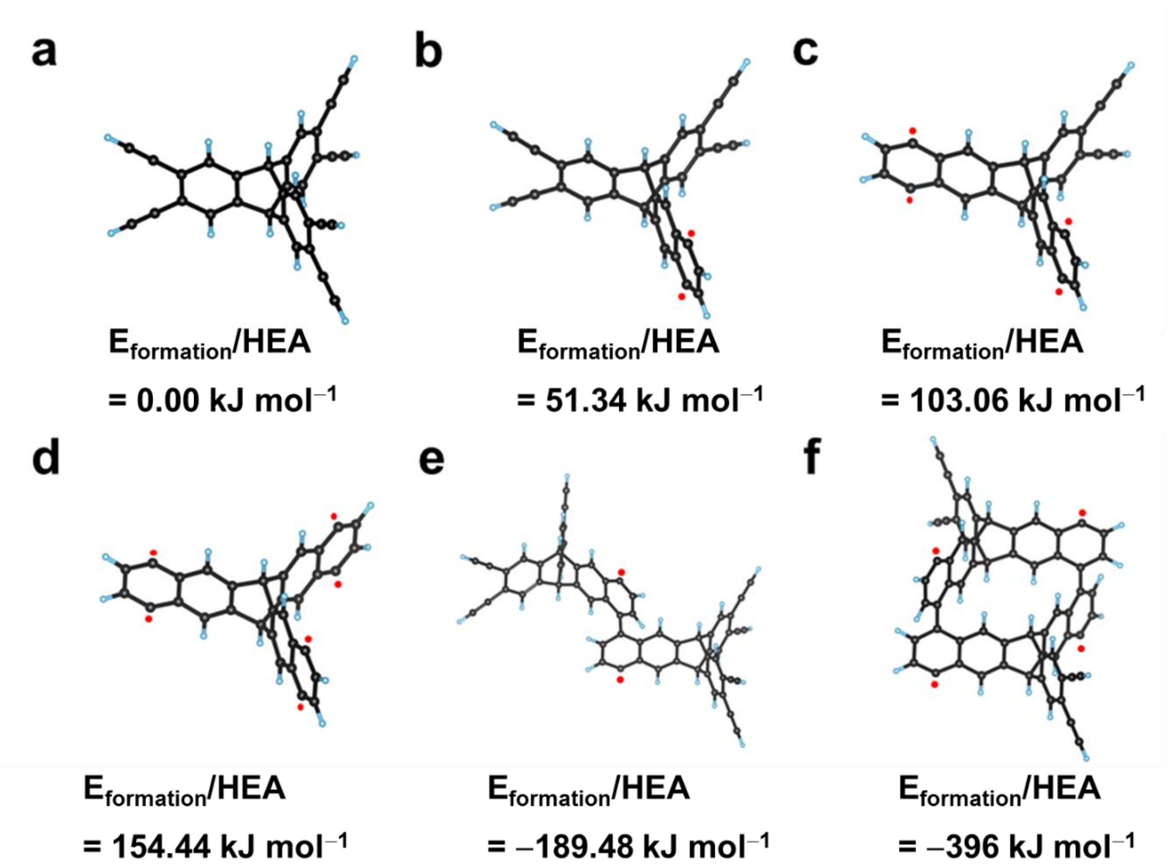

Supplementary Figure 5 | Energy of formation of different intermediates. a, The geometry of HEA single molecule. The geometries of polyHEA units after the Bergman reaction (cycloaromatization): **(b)** one ring; **(c)** two rings and **(d)** three rings. The bonding configuration after the Bergman reaction of two HEA molecules: **(e)** one ring and **(f)** two rings. The red dots in **(b), (c), (d), (e)** and **(f)** indicate the sp^2 carbon dangling bond states.

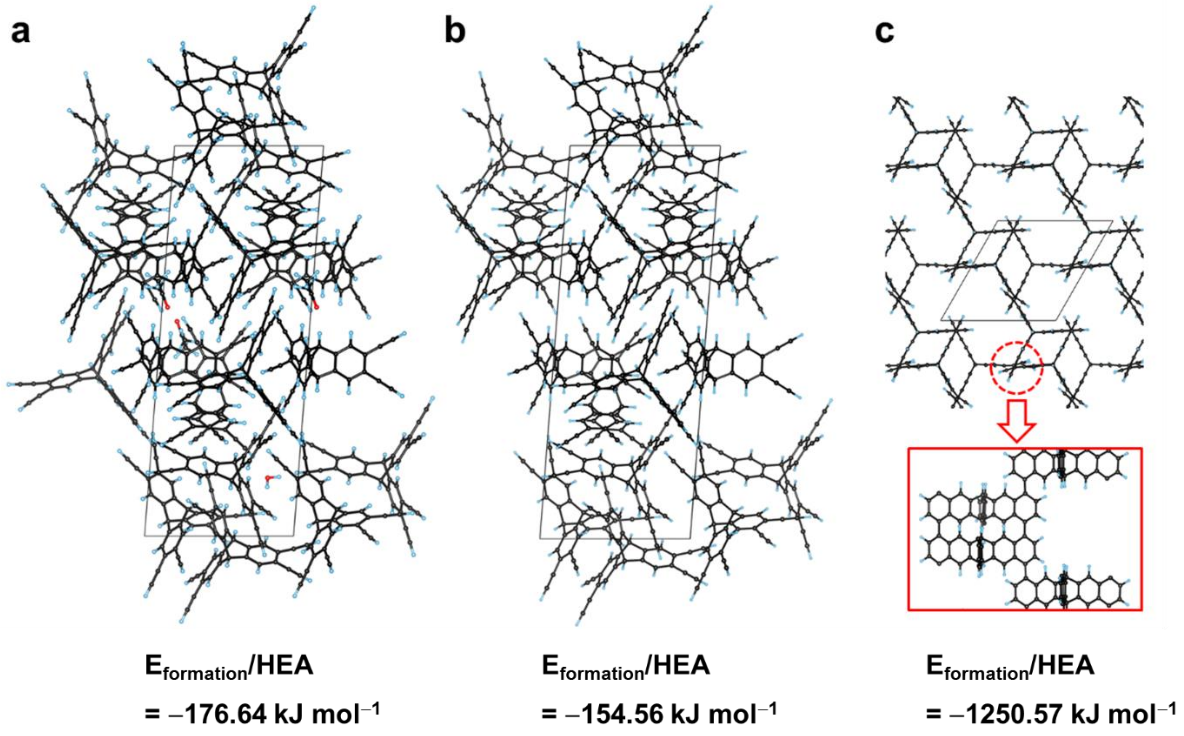

Supplementary Figure 6 | Formation energies evaluated by DFT calculations. a, HEA crystal lattice with two acetone and one water molecules (–176.64 kJ mol^{-1}); **b,** HEA crystal lattice without two acetone and one water molecules (–154.56 kJ mol^{-1}); **c,** cross-sectional view of the bulk crystal structure of polyHEA after the Bergman reaction of HEA molecules (–1250.57 kJ mol^{-1}); the closed up side view near the bond between two HEA molecules are presented in the red box below. The black solid boxes in **(a)**, **(b)** and **(c)** are periodic unit cell of solid. Color code: black, carbon; red, oxygen; cyan, hydrogen.

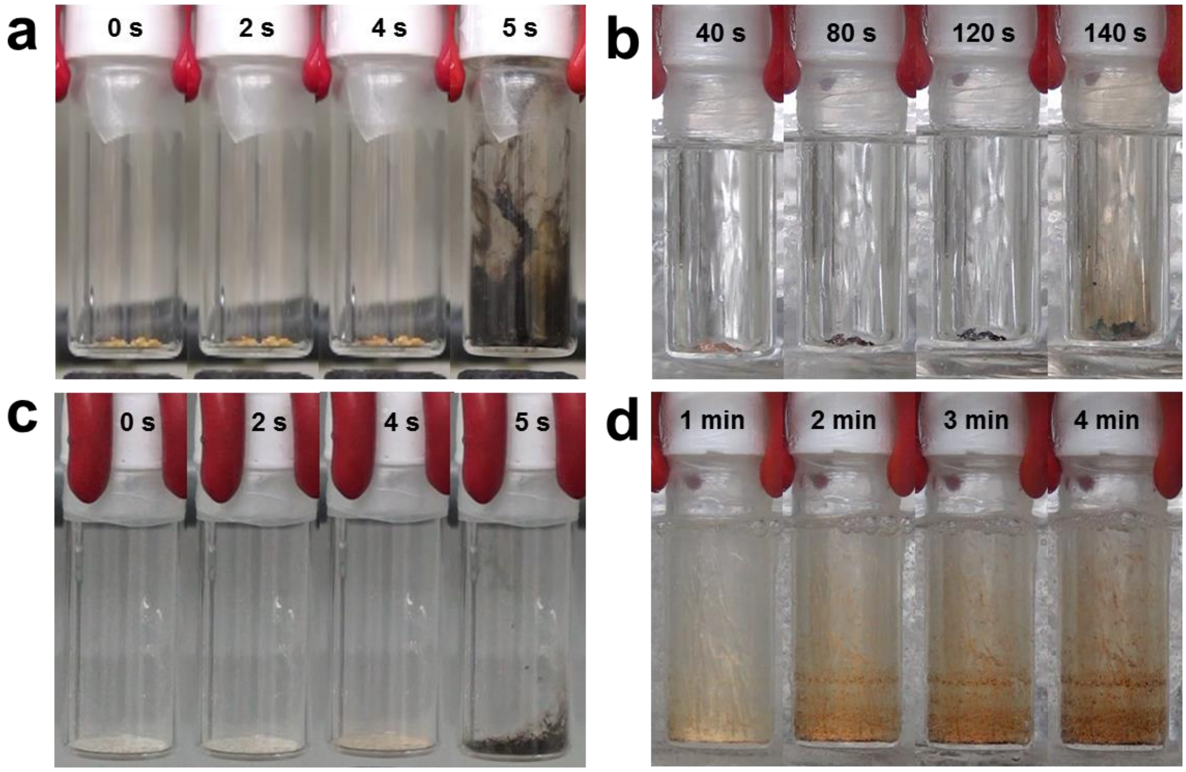

Supplementary Figure 7 | Additional control experiments using bulk and ground HEA crystals. Photographs of samples with respect to ramping rate of temperature under argon atmosphere. a, Bulk crystals heated by heat-gun with ramping rate of 23 °C s^{-1}; **b,** bulk crystals in oil-bath with ramping rate of 0.8 °C s^{-1}; **c,** ground crystals heated by heat-gun with ramping rate of 23 °C s^{-1}; **d,** ground crystals in oil-bath with ramping rate of 0.8 °C s^{-1}.

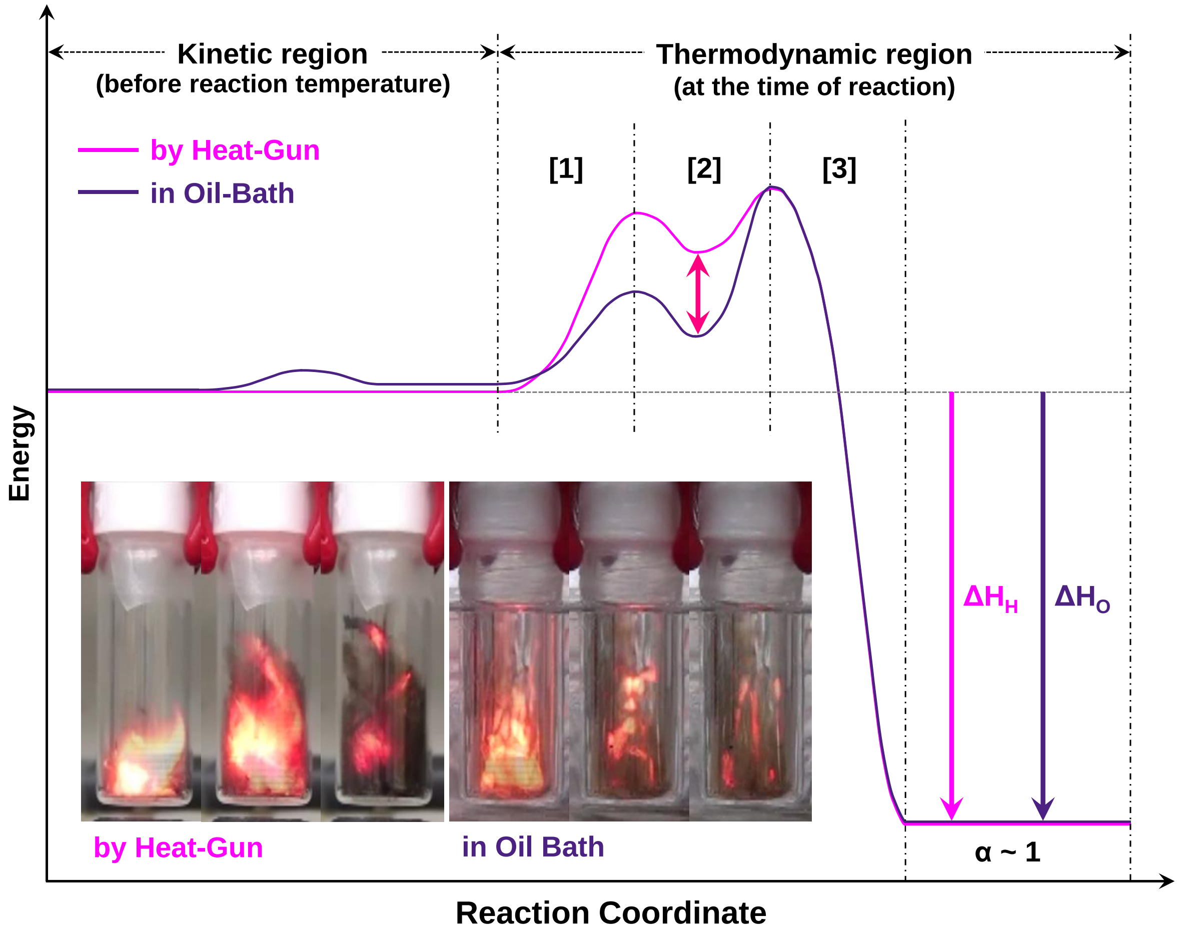

Supplementary Figure 8 | Energy diagrams of bulk crystals with different heating rate by heat-gun (rapid heating) and in oil-bath (slow heating). Insets are photographs of the moment of the explosive Bergman reaction induced by heat-gun and in oil-bath.

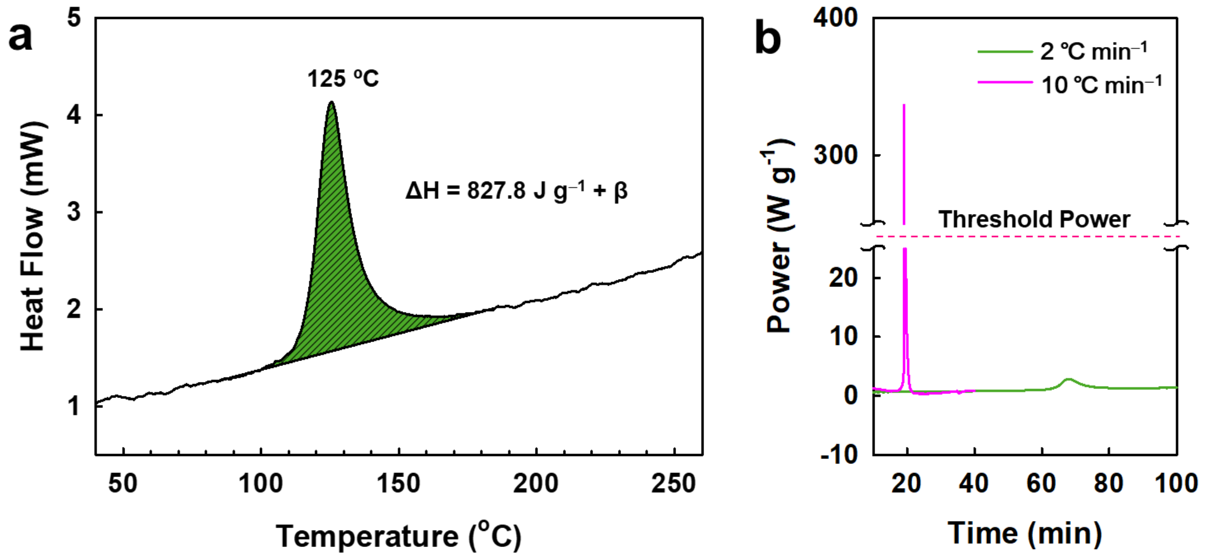

Supplementary Figure 9 | Thermal behaviour of the bulk HEA crystals from DSC measurement. a, DSC thermogram obtained from the 1st heating scan of bulk HEA crystals with a slow heating rate of 2 °C min^{-1}. In case of HEA crystals heated by 2 °C min^{-1}, the reaction temperature is approximately 12 °C lower than that by 10 °C min^{-1}, because slow heating allows enough time for sample to absorb enough heat. The similar phenomenon can be found in the literature (10 °C min^{-1} *vs.* 1 °C min^{-1} in TGA)[4]; **b,** DSC thermograms of bulk crystals showing amount of exothermic heat with respect to time (x-axis) at different heating rate. Upon rapid heating (10 °C min^{-1}), a sharp and strong exothermic peak at around 20 min (pink) implies that a large amount of heat releases during shorter period of time, while a broad and weak exothermic peak at around 70 min (green) indicates that a small amount of heat releases during longer period of time.

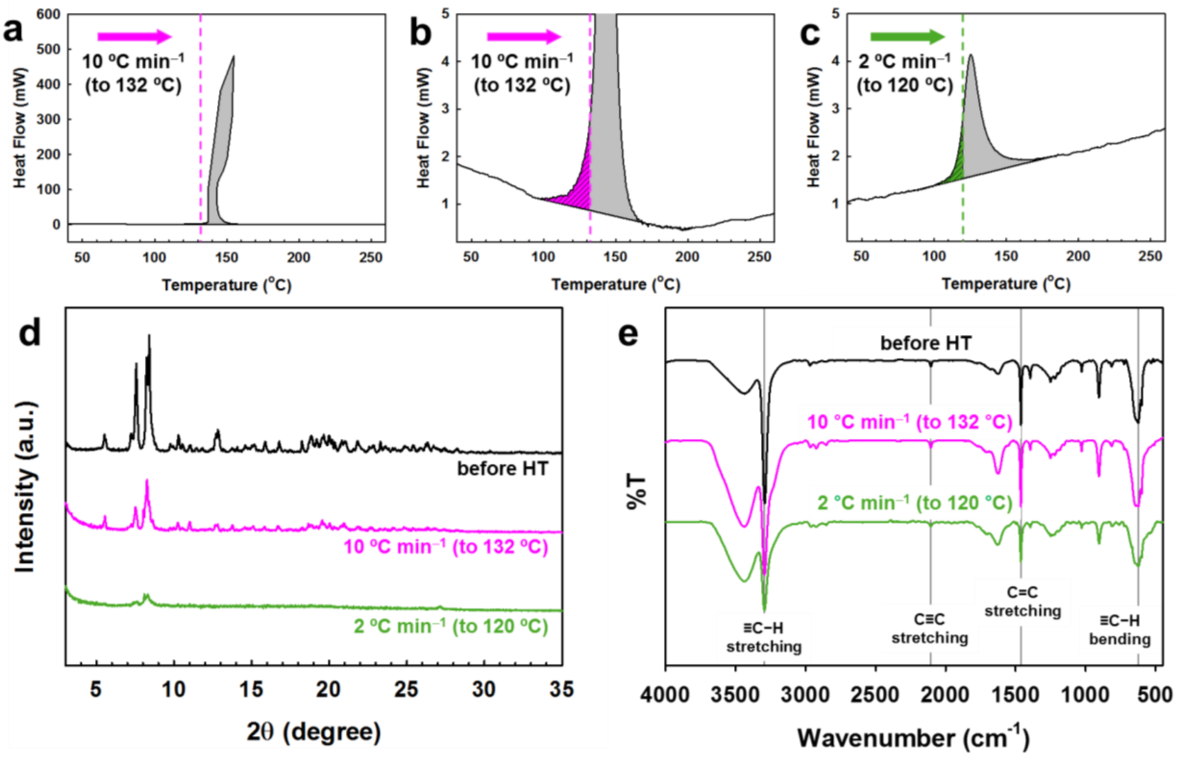

Supplementary Figure 10 | Heat-treatment below the reaction temperature of the bulk crystals using DSC. a, Heated to 132 °C, which is 5 °C lower than the reaction temperature at 137 °C with rapid ramping rate of 10 °C min^{-1}; **b,** magnification of y-axis of (**a**); **c,** heated to 120 °C, which is 5 °C lower than the reaction temperature at 125 °C with slow ramping rate of 2 °C min^{-1}. **d,** Powder XRD patterns of bulk crystals before (pristine) and after heat-treatments using DSC. **e,** FT-IR (KBr pellet) spectra of bulk crystals before (pristine) and after heat-treatments using DSC.

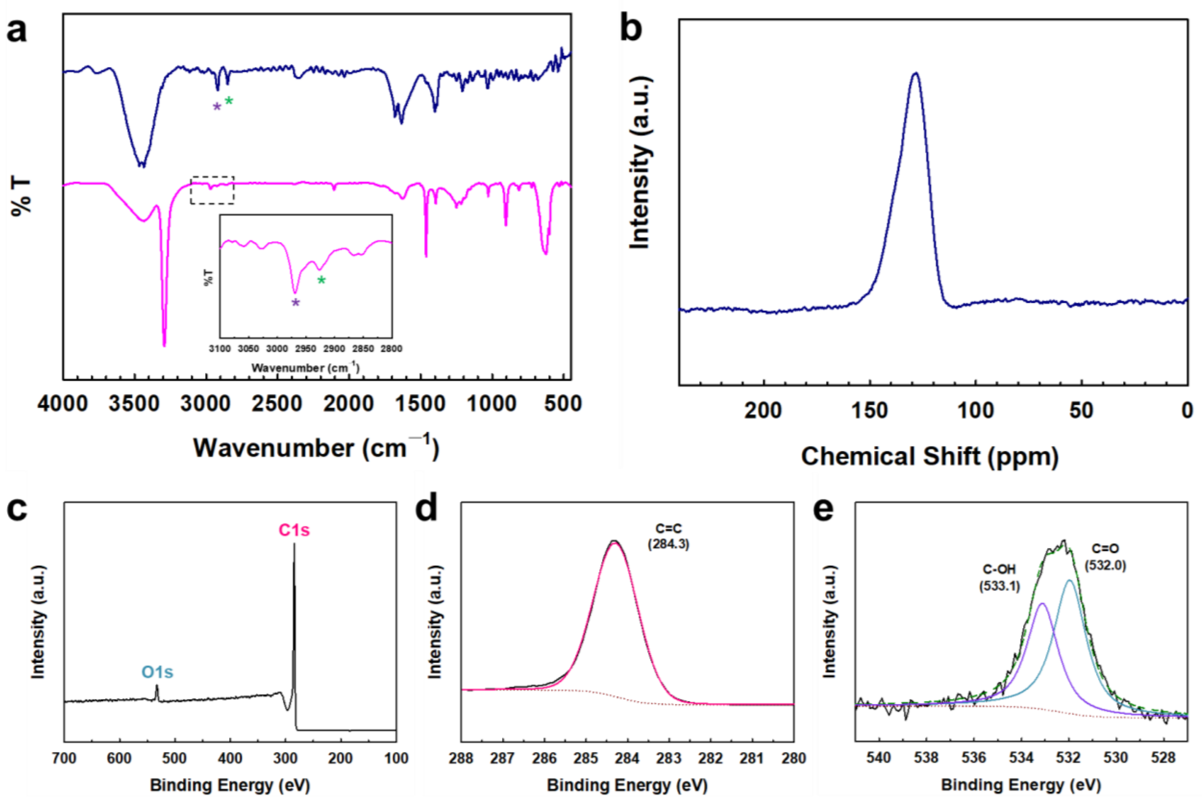

Supplementary Figure 11 | FT-IR, NMR and XPS analysis of the polyHEA and HEA. a, FT-IR (KBr pellet) spectra of polyHEA (blue) and HEA (pink). Inset is a magnification of the black dot rectangle area. **b,** Solid-state magic angle ^{13}C NMR spectrum of polyHEA. XPS survey spectra of polyHEA: **c,** Full spectrum; **d,** C 1s; **e,** O 1s.

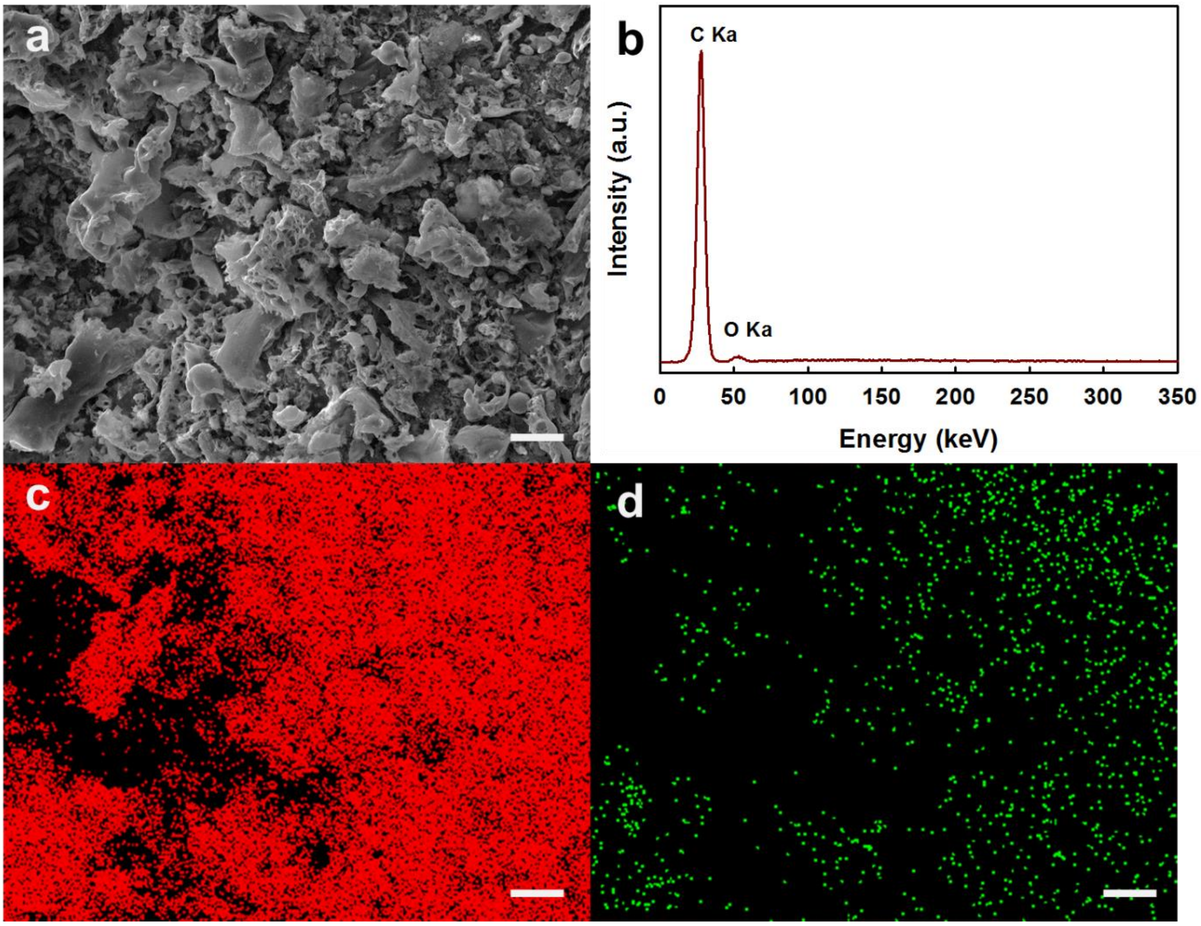

Supplementary Figure 12 | Elemental composition and distribution of elements from SEM-EDS. a, SEM image of polyHEA. **b,** EDS spectrum of polyHEA. The corresponding element mappings of image (**a**): **c,** Carbon and **d,** Oxygen. Scale bars are 20 µm.

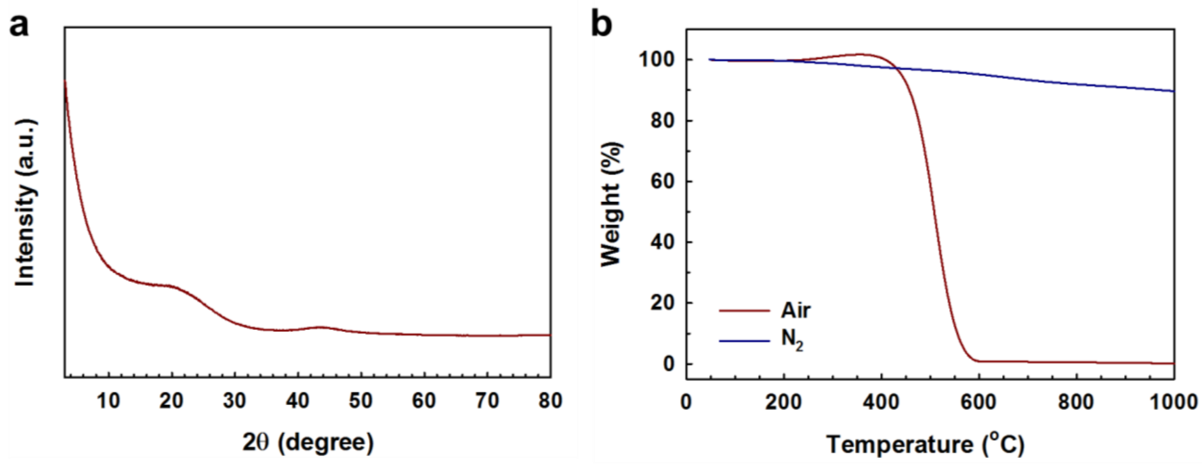

Supplementary Figure 13 | XRD and TGA behaviour of the polyHEA. a, Powder X-ray diffraction pattern of polyHEA. **b,** TGA thermograms of polyHEA obtained with ramping rate of 10 °C min^{-1} in air and nitrogen. Slight weight gaining in dry air atmosphere in the range of 250-450 °C is associated with gas (oxygen) uptake due to the porous nature of polyHEA.

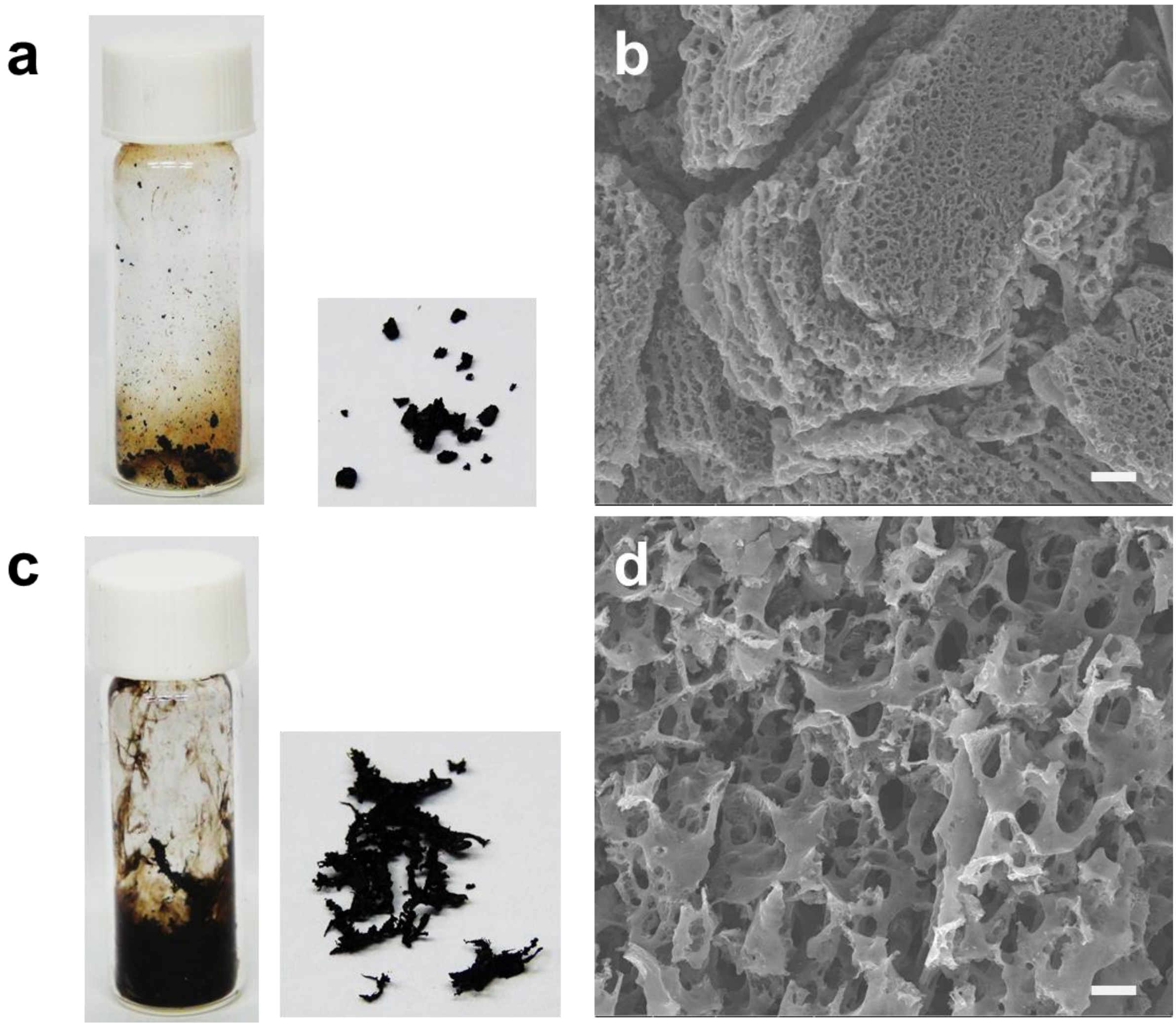

Supplementary Figure 14 | The reaction of bulk HEA crystals under slowly elevated temperature in oil-bath. a, Photograph and **b,** SEM image (Scale bar: 10 µm). **The reaction of bulk HEA crystals under rapidly elevated temperature using heat-gun. c,** Photograph and **d,** SEM image (Scale bar: 10 µm). The polyHEA obtained from slow heating in oil-bath shows more compact macropores than those obtained from rapid heating by heat-gun.

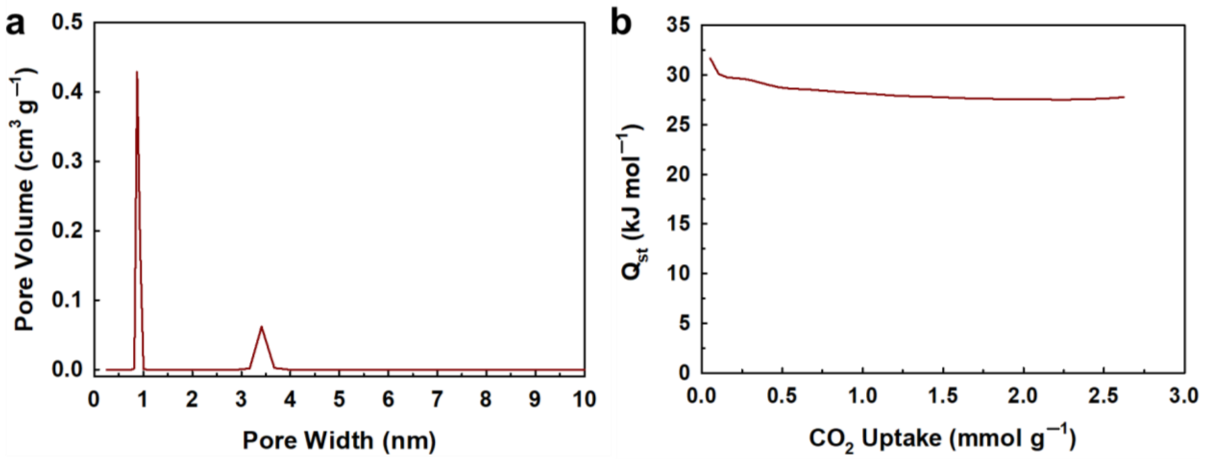

Supplementary Figure 15 | Pore-size distribution and heat of adsorption from the BET analysis. a, Pore-size distribution of polyHEA calculated by GCMC method. **b,** Isosteric heat of adsorption (Q_{st}) of polyHEA for CO_2 calculated from the adsorption curve at 273 K and 298 K.

Supplementary Table 1 | Crystal data and structure refinement for as-grown bulk HEA single crystals

Empirical formula	$C_{294}H_{140}O_3$
Formula weight	3720.05
Temperature	173(2) K
Wavelength	0.71073 Å
Crystal system	Triclinic
Space group	*P*1
Unit cell dimensions	a = 13.739(3) Å α = 80.76(3)°<br>b = 13.973(3) Å β = 78.58(3)°<br>c = 32.901(7) Å γ = 61.52(3)°
Volume	5426(2) $Å^3$
Z	1
Density (calculated)	1.139 Mgm^{-3}
Absorption coefficient	0.066 mm^{-1}
F(000)	1928
Crystal size	0.320 × 0.300 × 0.170 mm^3
Theta range for data collection	3.018 to 24.999°.
Index ranges	$-15 \le h \le 16$, $-16 \le k \le 16$, $-39 \le l \le 39$
Reflections collected	69913
Independent reflections	35246 [R(int) = 0.0597]
Completeness to theta = 24.999°	99.4 %
Absorption correction	Semi-empirical from equivalents
Max. and min. transmission	0.989 and 0.979
Refinement method	Full-matrix least-squares on F^2
Data / restraints / parameters	35246 / 216 / 2666
Goodness-of-fit on F^2	1.029
Final R indices [I>2sigma(I)]	R1 = 0.0889, wR2 = 0.2391
R indices (all data)	R1 = 0.1827, wR2 = 0.3278
Absolute structure parameter	4.5(10)
Largest diff. peak and hole	0.708 and –0.363 e·$Å^{-3}$

Supplementary Table 2 | Elemental composition of the polyHEA from different characterization techniques

Technique	C	H	O	Total
**Theoretical (wt%)**	96.46	3.54	0.00	100
**EA (wt%)[a]**	90.59	1.97	7.35	99.91
**XPS (at%)**	96.75	-	3.25	100
**SEM EDS (at%)**	96.61	-	3.39	100
**SEM EDS (wt%)**	95.53	-	4.47	100

[a] The oxygen content of polyHEA in elemental analysis (EA) is approximately 4 wt% higher than those from XPS and SEM EDS analyses, because the EA measurement is accomplished by combustion method. As shown in **Supplementary Fig. 13b**, the TGA thermogram of polyHEA demonstrates that mass gain occurs in range of about 250 °C to 450 °C in air atmosphere due to air (oxygen) adsorption.

Supplementary Table 3 | Comparison of surface area, CO_2 uptake (at 273 K, 1 bar) and isosteric heat (Q_{st}) of various PONs reported in the literatures

Porous materials	Nitrogen (wt%)	S_{BET} ($m^2 g^{-1}$)	CO_2 uptake (mmol g^{-1})	Q_{st} (kJ mol^{-1})	Ref.
polyHEA	–	1176	3.93	31.7	This work
Network-1	–	1980	3.63	23.3	5
Network-E	–	1470	2.95	25.5	6
Network-A	–	4077	2.65	23.0	6
Network-D	–	1213	2.42	26.0	6
BPL carbon	–	1150	2.09	–	6
PAF-1	–	5460	2.07	15.6	7
CMP-1	–	837	2.05	27.2	8
FCTF-1-600	15.4[a]	1535	5.53	35.0	9
BILP-4	14.0	1135	5.41	28.7	10
ALP-1	12.5	1235	5.36	29.2	11
BILP-3	9.96	1306	5.11	28.6	12
CPOP-1	7.32[b]	2220	4.82	27.0	13
Cz-POF-3	5.97	1927	4.77	27.8	14
APOP-3	29.5	1402	4.54	27.5	15
BILP-7	10.9	1122	4.39	27.8	10
TSP-2	7.92	913	4.1	30.2	16
Network-C	14.6	1237	3.86	33.0	6
SNW-1	29.9	821	3.64	35.0	17
PECONF-3	24.0[b]	851	3.49	26.0	18
STPI-2	7.12	541	3.34	36.0	19
TPI-1@IC	7.09	1053	3.22	49.3	20
NPTN-2	10.6	1558	3.18	37.0	21
NPTN-1	15.3	1187	3.01	34.0	21
Py-1	10.9	437	2.7	36.0	22
azo-COP-2	14.4	702.6	2.55	24.8	23
azo-COP-1	14.2	608.1	2.44	29.3	23
NPTN-3	8.24	1055	2.22	30.0	21

[a] Measured value from XPS (at%); [b] Calculated value from structure.

Supplementary Note 1

The density functional theory (DFT) of HEA single molecule was calculated by Vienna Ab initio Simulation Package[2]. To describe electronic structure, Perdew-Burke-Ernzerhof-type exchange correlation functional[3] and plane wave basis set with 400 eV energy cut-off are employed. The single gamma point of Brillouin zone is only sampled under the periodic boundary condition. The formation energy of the Bergman reaction (cycloaromatization) of HEA molecule was found to be substantially endothermic process, which costs, on average, 51.45 kJ mol^{-1} for single ring formation (**Supplementary Fig. 5a-5d**). Nevertheless, the presence of sp^2 carbon dangling bond states, as a result of the Bergman reaction in HEA molecule, provides strong deriving force toward the chain reaction to form polyHEA. For example, the formation of single C-C bond between two HEA molecules, as presented in **Supplementary Fig. 5e**, yields the energy gain of 189.48 kJ mol^{-1}, which overcomes the energy cost in the ring formation. This indicates that a consecutive bond formation between HEA molecules, through a chain reaction, can produce an enormous energy gain.

Supplementary Note 2

The crystallography data from single-crystal X-ray diffraction in **Fig. 1c** indicated that the pair of two acetone molecules and one water molecule were present per nine HEA molecules, which were preserved without either the cycloaromatization (aromatic ring formation). It is thought the presence of such gaseous species provided an amount of entropy, contributing to the reduction of the free energy of the lattice. We calculated the energy change using the density functional total energy minimization and found that that the evacuation of acetone and water molecules results in a small energy cost of

approximately 22.08 kJ mol^{-1} followed by volume reduction (**Supplementary Fig. 6a** and **6b**). Disappearance of the gaseous species can possibly contribute to the further increase in terms of free energy incurred by the reduction of entropy. However, the energy gain obtained by the formation of C-C bonds between two HEA molecules is much stronger. Hence, the overall chain reaction can lead to explosive exothermic reaction. There are diverse path of polymerizations. For example, as presented in **Supplementary Fig. 6c**, we calculated one model geometry of perfectly crystalized form, in which all covalently bonded HEA units constitute the three-dimensional (3D) porous organic network (PON). For this ideal formation of PON, the energy gain was found to be 1250.57 kJ mol^{-1}.

Supplementary Note 3

Control experiments of bulk and ground crystals with different heating rate. When the bulk crystals were rapidly heated by heat-gun (ramping rate of 23 °C s^{-1}), explosive reaction occurred (**Supplementary Fig. 7a**). Upon slow heating in oil-bath (ramping rate of 0.8 °C s^{-1}), the color of HEA crystals was gradually changed from yellow to black maintaining initial crystal morphology and weak explosive reaction also occurred (**Supplementary Fig. 7b**). Upon rapid heating of the ground crystals (**Supplementary Fig. 7c**), the explosive reaction occurred, but not as explosive as the bulk crystals (**Supplementary Fig. 7a**). This result implies that there is still enough change in lattice energy to drive explosive reaction even in the ground crystals. However, slow heating the ground crystals cannot abruptly release enough lattice energy in a short period of time to complete explosion as indicated by the color change stopped at deep orange (**Supplementary Fig. 7d**), which, in turn, manifests incomplete cycloaromatization.

Supplementary Note 4

Control experiments of bulk crystals with different heating rates. Interestingly, exothermic heat and kinetic energy of molecules at the moment of the Bergman reaction is quite different (**inset photographs, Supplementary Fig. 8, and frames 3 and 4 of Supplementary Video 1**). Instantaneously generated reaction heat upon rapid heating by heat-gun is much higher than that by slow heating in oil-bath. Such a phenomenon could be induced by the amount of energy at given time upon releasing primer molecules (acetone and water) in the bulk crystals (**stage 2, Supplementary Fig. 8**). As shown the energy diagrams in **Supplementary Fig. 8**, the bulk crystals in oil-bath take longer time for explosive reaction triggered by abrupt evacuation of primer molecules, and therefore kinetic energy generated by primer molecules significantly reduced at the moment of reaction.

Supplementary Note 5

Control experiments of bulk crystals with different heating rates (amount of heat per given time). To prove the hypothesis (explosive reaction induced by primer molecules), as-grown bulk HEA crystals was scanned at very slow ramping rate of 2 °C min^{-1}, allowing enough time for the gradual release of primer molecules for longer period of time. In this case, explosive solid-state Bergman reaction did not occur, because of insufficient energy per given time to overcome activation barrier (**Supplementary Fig. 9a**).

Supplementary Note 6

Control experiments of bulk crystals with different heat-treatment conditions (annealing time dependence). To further support the proposed mechanism for explosive reaction induced by primer molecules, the bulk HEA crystals were heated to 132 °C with ramping rate

of 10 °C min^{-1} (**Supplementary Fig. 10a and 10b**) and 120 °C with ramping rate of 2 °C min^{-1} (**Supplementary Fig. 10c**), respectively, to the temperature 5 °C lower than the reaction temperature (137 °C and 125 °C). In order to check the structural and chemical changes before and after heat-treatments, powder XRD patterns (**Supplementary Fig. 10d**) and FT-IR spectra (**Supplementary Fig. 10e**) were obtained. The bulk HEA crystals were not induced explosive reaction in both cases of rapid (10 °C min^{-1}) and slow (2 °C min^{-1}) heating. The crystallinity of slowly heated sample is substantially reduced due to the evacuation of most acetone and water molecules, while the rapid heated sample still maintains significant degree of crystallinity (**Supplementary Fig. 10d**). However, FT-IR spectra of the samples before and after heat-treatments are similar each other (**Supplementary Fig. 10e**), implying that there is only change in crystallinity but no change in chemical identity. On the basis of this scenario, sufficient lattice energy change at given period of time is necessary to overcome threshold point for structural rearrangement of HEA molecules and thus induces explosive Bergman reaction of enediyne groups in their solid-state (**Supplementary Fig. 9b**).

Supplementary Methods

Materials. All the solvents, chemicals and reagents were purchased from Aldrich Chemical Inc., unless otherwise stated. Anhydrous diisopropylamine (i-Pr_2NH) was freshly prepared from distillation of commercial i-Pr_2NH in the presence of calcium hydride (CaH_2).

Instrumentations. X-ray diffraction (XRD) patterns were taken on a High Power X-Ray Diffractometer, Rigaku, Japan and a Single-Crystal X-ray diffractometer R-AXIS RAPID II, Rigaku, Japan. Differential scanning calorimetry (DSC) was recorded on a TA, Q200. Solid-state magic angle spinning (MAS) carbon thirteen (^{13}C) nuclear magnetic resonance (NMR) spectrum was taken on a 600 MHz FT-NMR, WNMRS 600, Agilent. Fourier transform infrared (FT-IR) spectra were recorded on a Perkin-Elmer Spectrum 100 using KBr disks. X-ray photoelectron spectroscopy (XPS) was performed on an X-ray Photoelectron Spectroscopy Thermo Fisher K-alpha (UK). Scanning electron microscope (SEM) images were taken on a Field Emission Scanning Electron Microscope Nanonova 230 FEI, USA. Transmission electron microscopy (TEM) was performed using a JEM-2100 (JEOL, Japan) under an operating voltage of 200 keV. The specimens for TEM were prepared by drop casting of sample dispersed in acetone on Quantifoil holey carbon TEM grid and dried in oven at 80 °C. The specific surface area was measured by nitrogen adsorption-desorption isotherms using the Brunauer-Emmett-Teller (BET) method on a Micromeritics ASAP 2504N. Element analysis (EA) was performed on a Flash 2000, Thermo. Themogravimetric analysis (TGA) was conducted using a Thermogravimetric AnalyzerQ200 TA Instrument, USA.

Crystallographic data collection and refinement of the structure. A crystal of as-grown bulk HEA single crystals was coated with paratone oil and the diffraction data measured at 173 K

with Mo Kα radiation on an X-ray diffraction camera system using an imaging plate equipped with a graphite crystal incident beam mono chromator. The Rapid Auto software[24] was used for data collection and data processing. Structure was solved by direct method and refined by full-matrix least-squares calculation with the SHELX software package[24].

Nine HEAs, two acetone and one water molecules are observed as an asymmetric unit. All non-hydrogen atoms are refined anisotropically; the hydrogen atoms were assigned isotropic displacement coefficients U (H) = 1.2U (C) and 1.5U (C_{methyl}), their coordinates were allowed to ride on their respective atoms. The least-squares refinement of the structural model was performed under geometry restraints and displacement parameter restraint such as DANG, DFIX, DELU, ISOR and SIMU.

Refinement of the structure converged at a final $R1$ = 0.0889 and $wR2$ = 0.2391 for 16856 reflections with $I > 2\sigma(I)$; $R1$ = 0.1827 and $wR2$ = 0.3278 for all 35246 reflections. The largest difference peak and hole were 0.708 and –0.363 e·$Å^{-3}$, respectively.

A summary of the crystal and some crystallography data is given in Supplementary Table 1. CCDC-1475255 contains the supplementary crystallographic data for this paper. The data can be obtained free of charge at www.ccdc.cam.ac.uk/conts/retrieving.html or from the Cambridge Crystallographic Data Centre, 12, Union Road, CambridgeCB2 1EZ, UK.

Supplementary References

1. Sheldrick, G. Crystal structure refinement with SHELXL. *Acta Crystallogr. C* **71**, 3–8 (2015).
2. Kresse, G. & Furthmüller, J. Efficiency of ab-initio total energy calculations for metals and semiconductors using a plane-wave basis set. *Comput. Mater. Sci.* **6**, 15–50 (1996).
3. Perdew, J. P., Burke, K. & Ernzerhof, M. Generalized gradient approximation made simple. *Phys. Rev. Lett.* **77**, 3865–3868 (1996).
4. Bae, S.-Y. *et al.* Large-area graphene films by simple solution casting of edge-selectively functionalized graphite. *ACS Nano* **5**, 4974–4980 (2011).
5. Yao, S., Yang, X., Yu, M., Zhang, Y. & Jiang, J.-X. High surface area hypercrosslinked microporous organic polymer networks based on tetraphenylethylene for CO_2 capture. *J. Mater. Chem. A* **2**, 8054–8059 (2014).
6. Dawson, R., Stockel, E., Holst, J. R., Adams, D. J. & Cooper, A. I. Microporous organic polymers for carbon dioxide capture. *Energy Environ. Sci.* **4**, 4239–4245 (2011).
7. Ben, T. *et al.* Gas storage in porous aromatic frameworks (PAFs). *Energy Environ. Sci.* **4**, 3991–3999 (2011).
8. Dawson, R., Adams, D. J. & Cooper, A. I. Chemical tuning of CO_2 sorption in robust nanoporous organic polymers. *Chem. Sci.* **2**, 1173–1177 (2011).
9. Zhao, Y., Yao, K. X., Teng, B., Zhang, T. & Han, Y. A perfluorinated covalent triazine-based framework for highly selective and water-tolerant CO_2 capture. *Energy Environ. Sci.* **6**, 3684–3692 (2013).
10. Rabbani, M. G. & El-Kaderi, H. M. Synthesis and characterization of porous benzimidazole-linked polymers and their performance in small gas storage and selective uptake. *Chem. Mater.* **24**, 1511–1517 (2012).

11. Arab, P., Rabbani, M. G., Sekizkardes, A. K., İslamoğlu, T. & El-Kaderi, H. M. Copper(I)-catalyzed synthesis of nanoporous azo-linked polymers: Impact of textural properties on gas storage and selective carbon dioxide capture. *Chem. Mater.* **26**, 1385–1392 (2014).

12. Rabbani, M. G., Reich, T. E., Kassab, R. M., Jackson, K. T. & El-Kaderi, H. M. High CO_2 uptake and selectivity by triptycene-derived benzimidazole-linked polymers. *Chem. Commun.* **48**, 1141–1143 (2012).

13. Chen, Q. *et al.* Microporous polycarbazole with high specific surface area for gas storage and separation. *J. Am. Chem. Soc.* **134**, 6084–6087 (2012).

14. Zhang, X., Lu, J. & Zhang, J. Porosity enhancement of carbazolic porous organic frameworks using dendritic building blocks for gas storage and separation. *Chem. Mater.* **26**, 4023–4029 (2014).

15. Song, W.-C., Xu, X.-K., Chen, Q., Zhuang, Z.-Z. & Bu, X.-H. Nitrogen-rich diaminotriazine-based porous organic polymers for small gas storage and selective uptake. *Polym. Chem.* **4**, 4690–4696 (2013).

16. Zhu, X. *et al.* Efficient CO_2 capture by a task-specific porous organic polymer bifunctionalized with carbazole and triazine groups. *Chem. Commun.* **50**, 7933–7936 (2014).

17. Gao, X., Zou, X., Ma, H., Meng, S. & Zhu, G. Highly selective and permeable porous organic framework membrane for CO_2 capture. *Adv. Mater.* **26**, 3644–3648 (2014).

18. Mohanty, P., Kull, L. D. & Landskron, K. Porous covalent electron-rich organonitridic frameworks as highly selective sorbents for methane and carbon dioxide. *Nat. Commun.* **2**, 401 (2011).

19. Zhang, C. *et al.* Triptycene-based microporous polyimides: Synthesis and their high selectivity for CO_2 capture. *Polymer* **55**, 3642–3647 (2014).

20. Wu, S. *et al.* A rational construction of microporous imide-bridged covalent-organic polytriazines for high-enthalpy small gas absorption. *J. Mater. Chem. A* **3**, 878–885 (2015).

21. Wu, S. *et al.* Facile preparation of dibenzoheterocycle-functional nanoporous polymeric networks with high gas uptake capacities. *Macromolecules.* **47**, 2875–2882 (2014).

22. Luo, Y., Li, B., Wang, W., Wu, K. & Tan, B. Hypercrosslinked aromatic heterocyclic microporous polymers: A new class of highly selective CO_2 capturing materials. *Adv. Mater.* **24**, 5703–5707 (2012).

23. Patel, H. A. *et al.* Unprecedented high-temperature CO_2 selectivity in N_2-phobic nanoporous covalent organic polymers. *Nat. Commun.* **4**, 1357 (2013).

24. Rapid Auto software, R-Axis series, Cat. No. 9220B101, Rigaku Corporation.
