# Supplementary material for: Forming a three-dimensional porous organic network via solid-state explosion of organic single crystals
Source: Nat Commun. 2017 Nov 17;8:1599. doi: 10.1038/s41467-017-01568-3 (PMC5693943; doi:10.1038/s41467-017-01568-3)
Supplement: Supplementary file 3 — Description of Additional Supplementary File [file 41467_2017_1568_MOESM3_ESM.pdf]

Description of Additional Supplementary Files

File Name: Supplementary Movie 1
Description: **Four experiments for the solid-state Bergman reaction at different conditions.** Each experiment contains normal and slow speed plays, showing a minor ignition followed by a major explosion.
